# The complete chloroplast genome sequence of *Anogeissus acuminata* (combretaceae)

**DOI:** 10.1080/23802359.2020.1756958

**Published:** 2020-05-12

**Authors:** Jiaojun Yu, Tongmei Xu, Hongyu Wang, Dange Chen, Hongjin Dong

**Affiliations:** a Hubei Key Laboratory of Economic Forest Germplasm Improvement and Resources Comprehensive Utilization, Huanggang Normal University, Huanggang, China; b Hubei Collaborative Innovation Center for the Characteristic Resources Exploitation of Dabie Mountains, Huanggang, China; c Pu’er Forestry and Grassland Bureau, Pu’er, China

**Keywords:** *Anogeissus*, complete chloroplast genome, Combretaceae, phylogeny

## Abstract

*Anogeissus acuminata* (Roxburgh ex Candolle) Guillemin et al. is an Endangered and dominant species of deciduous forests distributed in the Mekong valley of southwest China and adjacent Indo-China Peninsula. Here we assembled and annotated the complete chloroplast (cp) genome. It is 159,993 bp in length and encodes 84 protein-coding genes, 37 transfer RNA (tRNA) genes and eight ribosomal RNA (rRNA) genes. This chloroplast genome sequencing offers genetic background for conservation and phylogenetic studies.

*Anogeissus* is a small genus distributed in tropical Africa and Asia, only one species occurs in China *A. acuminata* is edemic to tropical Asia, as a dominant tree deciduous forests distributed in the Mekong valley of southwest China and adjacent Indo-China Peninsula under 700 m in altitudes (Chen and Turland 2007).

The species was listed as Endangered in China by Shun (Fu 1992). In the present study, the completed chloroplast genome sequence of *A. acuminata* is reported contributing for better understanding its evolution and population genetics, and also providing significant information for the phylogeny of Combretaceae.

Genomic DNA was extracted from fresh leaves of a seedling of *A. acuminata* from Mengkuang, Nuozhadu, Lancang, Yunnan, China (100°31′1.14″E, 22°29′58.50″N, 651 m, in the valley; *Xu Tongmei* et al.*19090*, 2019-1-29; KUN), the total genomic DNA was isolated according to a modified CTAB method (Doyle and Doyle 1987). Those leaves were stored in the refrigerator at -80 °C. Total genome DNA of *A. acuminata* was sequenced by Illumina Hiseq 2500 Sequencing System (Illumina, Hayward, CA, USA) to construct the shotgun library and assembled through the NOVOPlasty software (Dierckxsens et al. 2017).The low quality sequences were filtered out Using CLC Genomics Workbench v8.0 (CLC Bio, Aarhus, Denmark) and then reconstructed the chloroplast genome by using MITObim v1.8 (University of Oslo, Oslo, Norway; Kaiseraugst, Switzerland) (Hahn et al. 2013). The complete chloroplast genome of *A. acuminata* was annotated in Geneious ver. 10.1 (http://www.genei ous.com, Kearse et al. 2012) and then submitted to GenBank (accession no. MT242598). The genome annotation was performed by aligning with the cp genomes of relatively related species.

The size of chloroplast genome of *A. acuminata* is 159,993 bp, including a large single-copy (LSC) region of 88,430 bp and a small single-copy (SSC) region of 18,871 bp separated by a pair identical inverted repeat regions (IRs) of 26,346 bp each. A total of 129 genes were successfully annotated containing 84 protein-coding genes, 37 tRNA genes and 8 rRNA genes. GC content of the whole genome, IRs, LSC and SSC regions are 37.0, 43.0, 34.7 and 30.7%, respectively. GC content of IRs region is the highest. Twenty genes contain one intron, while 3 genes have two introns. The complete chloroplast genome sequence of *A. acuminata* and other species from Combretaceae were used to construct phylogenetic tree (Figure 1). The sequences were initially aligned using MAFFT (Katoh and Standley 2013) and then visualized and manually adjusted using BioEdit (Hall 1999). Take the plastome of *Oenothera biennis* (GenBank: NC_010361) as an out-group, a maximum likelihood analysis was performed with RAxML version 8 program (Alexandros 2014) using 1000 bootstrap. The result shows the position of *A. acuminata* from Myrtales, which is consistent with previous molecular results (Conti et al.^1997^; Bayly et al. 2013; Gu et al. 2019).

**Figure 1.**
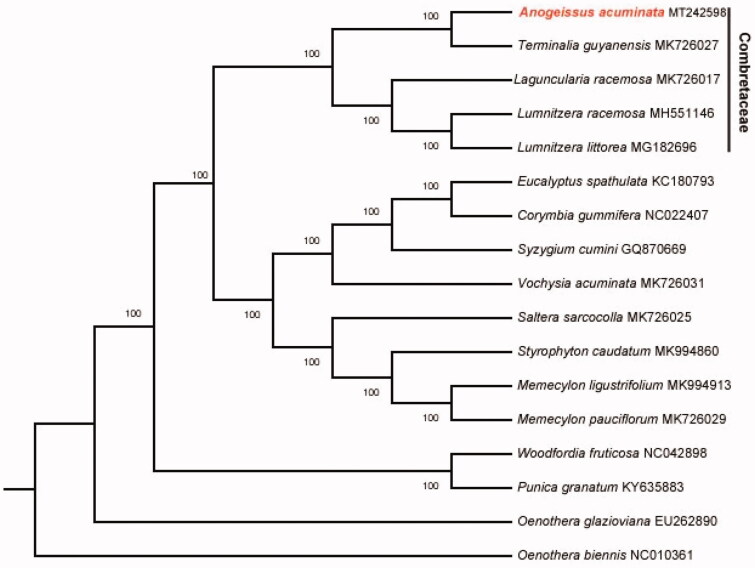
Maximum-likelihood phylogenetic tree for *A. acuminata* based on 16 complete chloroplast genomes. The number on each node indicates bootstrap support value.

## Data Availability

The data that newly obtained at this study are available in the NCBI under accession number of MT242598.
